# Fabrication of TiO_2_ Nanotubes Through Electrochemical Anodization and Secondary Oxidation

**DOI:** 10.3390/mi17091016

**Published:** 2026-08-27

**Authors:** Liheng Gao, Peihuan Li, Omer Farooq, Fubin Ma, Weijia Guo, Tianfeng Zhou

**Affiliations:** 1 School of Mechanical Engineering, Beijing Institute of Technology, Beijing 100081, China; 2 China Quality Certification Center Co., Ltd., Beijing 100700, China; 3 Beijing Institute of Precision Mechatronics and Controls, Beijing 100076, China; 4 State Key Laboratory of Chips and Systems for Advanced Light Field Display, Beijing Institute of Technology, Beijing 100081, China

**Keywords:** TiO_2_, anodization, nanotubes, oxidation, electrochemistry

## Abstract

Titanium dioxide nanotubes (TiO_2_ NTs) fabricated by electrochemical anodization have attracted attention because their morphology can be regulated by processing parameters. Although the anodic fabrication of TiO_2_ NTs has been widely studied, the morphology evolution of nanotubes on Ti6Al4V during secondary anodization after ultrasonic removal of the first nanotube layer still requires further clarification. In this study, anodic nanotubular oxide structures were fabricated on Ti6Al4V substrates by primary anodization and secondary anodization. The effects of anodization voltage, fluoride ion concentration, oxidation time, and secondary anodization on nanotube morphology were investigated. The results show that increasing anodization voltage promotes nanotube formation and increases tube diameter, whereas excessive oxidation time and high fluoride concentration lead to nanograss formation, tube collapse, and surface damage. Compared with primary anodization, secondary anodization produced smaller nanotube diameters and distinct morphology evolution, which may be associated with changes in the initial surface state after ultrasonic removal of the first nanotube layer. Among the investigated conditions, primary anodization at 40 V in 0.5 wt.% NH_4_F electrolyte followed by secondary anodization at 50 V produced relatively regular nanotubular regions, although nanograss was also present on the surface. This work provides a process-oriented understanding of TiO_2_ nanotube morphology regulation on Ti6Al4V substrates during primary and secondary anodization.

## 1. Introduction

Titanium dioxide nanotubes (TiO_2_ NTs), known for their high surface area and chemical stability, have attracted significant attention for applications in environmental remediation and energy conversion [1,2,3]. TiO_2_ NTs are nanostructured TiO_2_, distinguished by their tubular shape. These nanotubes are typically fabricated on titanium substrates via electrochemical anodization, a technique that enables the precise control of processing parameters to achieve certain morphological characteristics [4,5,6]. The nanostructures are well-known for their significant specific surface area and highly ordered structure, which are attributes that enhance their effectiveness in applications such as photocatalytic degradation and energy storage [7,8,9]. Moreover, the capacity to alter these nanotubes, for instance, by introducing additional elements, broadens their range of functions, rendering them appropriate for many applications in the fields of health, energy, and biosensing [10,11,12]. Importantly, electrochemical anodization is considered a cost-effective and scalable technique, which is highly relevant for industrial fabrication and surface engineering applications.

The investigation of TiO_2_ NTs has become a crucial domain in nanotechnology due to their applications in biomedical devices, environmental remediation, and energy conversion [13,14,15]. TiO_2_ nanostructures, predominantly in the form of nanotubes, have demonstrated promising performance in various technological applications [16,17]. Their potential in the biomedical field is reflected in their ability to enhance osteoblastic cell activity and improve corrosion resistance in bio functional implants [18,19]. Moreover, their applications in photocatalysis, supercapacitors, and solar cells highlight their versatility in addressing current technological and environmental challenges [20,21]. However, despite these advances, most studies primarily emphasize applications and functional performance, with comparatively less attention given to the detailed control of fabrication parameters during electrochemical anodization.

The electrochemical anodization process of TiO_2_ NTs has been extensively studied, particularly in terms of fabrication, modification, and practical applications [22,23]. Numerous studies have examined the production and modification of TiO_2_ NTs for applications such as photocatalytic water splitting. For example, electrochemical anodization has been applied for the degradation of methyl orange, demonstrating its effectiveness in water purification [24]. The study demonstrated the efficacy of this method for purifying water. Another study has explored the application of TiO_2_ nanotube arrays in photoelectrocatalytic degradation processes, highlighting that these arrays are commonly fabricated via electrochemical anodization in fluoride-containing electrolytes, which enables the formation of well-ordered nanotubular structures with enhanced catalytic performance [25,26]. The application of TiO_2_ nanotube coatings on titanium substrates has further demonstrated their potential in enhancing osseointegration and promoting bone regeneration, highlighting their significance in biomedical implant applications [27,28]. These studies indicate that anodization parameters, including voltage, electrolyte composition, and oxidation time, play a critical role in determining nanotube morphology and functional performance. Previous studies have also systematically investigated the effects of anodization voltage, electrolyte composition, fluoride concentration, oxidation time, and two-step anodization on the morphology regulation of TiO_2_ nanotubes [29,30,31,32].

Recent studies have further expanded the understanding of TiO_2_ nanotube systems, particularly in biomedical and surface engineering applications. Zhan et al. [33] demonstrated that tantalum-coated TiO_2_ nanotubes can significantly enhance sustained drug release performance and osteoinductive properties, highlighting the potential of nanotube modification strategies for advanced implant applications. Pathak et al. [34] investigated bioinspired nanocomposites for orthopedic applications, emphasizing the importance of mechanical behavior and structural optimization in nanostructured materials. In addition, Zhao et al. [35] reported that TiO_2_ nanofilms deposited on titanium implants can effectively reduce particle release, thereby improving implant stability and biocompatibility. Durdu et al. [36] examined the surface characteristics and antibacterial performance of well-ordered TiO_2_ nanotube arrays, demonstrating their effectiveness in preventing bacterial adhesion. Furthermore, recent reviews and studies have highlighted the role of nanotube-based surface modifications in improving osseointegration and corrosion resistance of titanium implants [37,38]. These findings indicate that tailoring nanotube structure and surface properties plays a critical role in enhancing functional performance across biomedical applications. Despite these advancements, most existing studies primarily focus on surface modification, biological performance, or application outcomes, with limited attention given to the detailed control of fabrication parameters during electrochemical anodization. In particular, the influence of secondary oxidation on nanotube growth behavior, structural stability, and morphological evolution remains insufficiently understood. Moreover, the interaction between anodization parameters and subsequent oxidation processes has not been systematically investigated, which limits precise control over nanotube architecture and hinders the optimization of fabrication processes for scalable and reproducible applications. Recent studies from Popat and co-workers have further highlighted the importance of TiO_2_ nanotube surface engineering and functionalization for improving the biological performance of titanium-based materials [39,40,41].

In this study, TiO_2_ nanotubes were fabricated on Ti6Al4V substrates by electrochemical anodization and secondary anodization after ultrasonic removal of the first nanotube layer. The influence of anodization voltage, fluoride ion concentration, and oxidation time on nanotube morphology was investigated. In addition, the morphology evolution during secondary anodization was analyzed by comparing primary and secondary anodized samples. The present work does not aim to claim the first demonstration of TiO_2_ nanotube anodization or functional superiority of the secondary-anodized samples. Instead, the specific contribution of this work is the direct comparison of primary and secondary anodization on Ti6Al4V after ultrasonic removal of the first nanotube layer, together with numerical analysis of the associated local electrochemical and mass-transfer behavior. The results provide a process-oriented understanding of the morphology evolution of anodic nanotubes during secondary anodization on Ti6Al4V.

## 2. Materials and Methods

### 2.1. Experimental Details

Medical-grade Ti6Al4V alloy (Baoji Titanium Industry Co., Ltd., Baoji, China) conforming to GB/T 3620.1-2007 [42] was used as the substrate. Before anodization, the specimens were sequentially ground using 600-, 800-, and 2000-grit abrasive papers (Struers ApS, Ballerup, Denmark), followed by polishing with diamond abrasive (Struers ApS, Ballerup, Denmark) on a velvet cloth. The specimens were then ultrasonically cleaned in acetone (analytical grade, Sinopharm Chemical Reagent Co., Ltd., Shanghai, China), absolute ethanol (analytical grade, Sinopharm Chemical Reagent Co., Ltd., Shanghai, China), and deionized water (prepared in-house) for 20 min each and naturally dried. After pretreatment, the surface roughness was approximately Ra = 60 nm.

**Figure 1:**
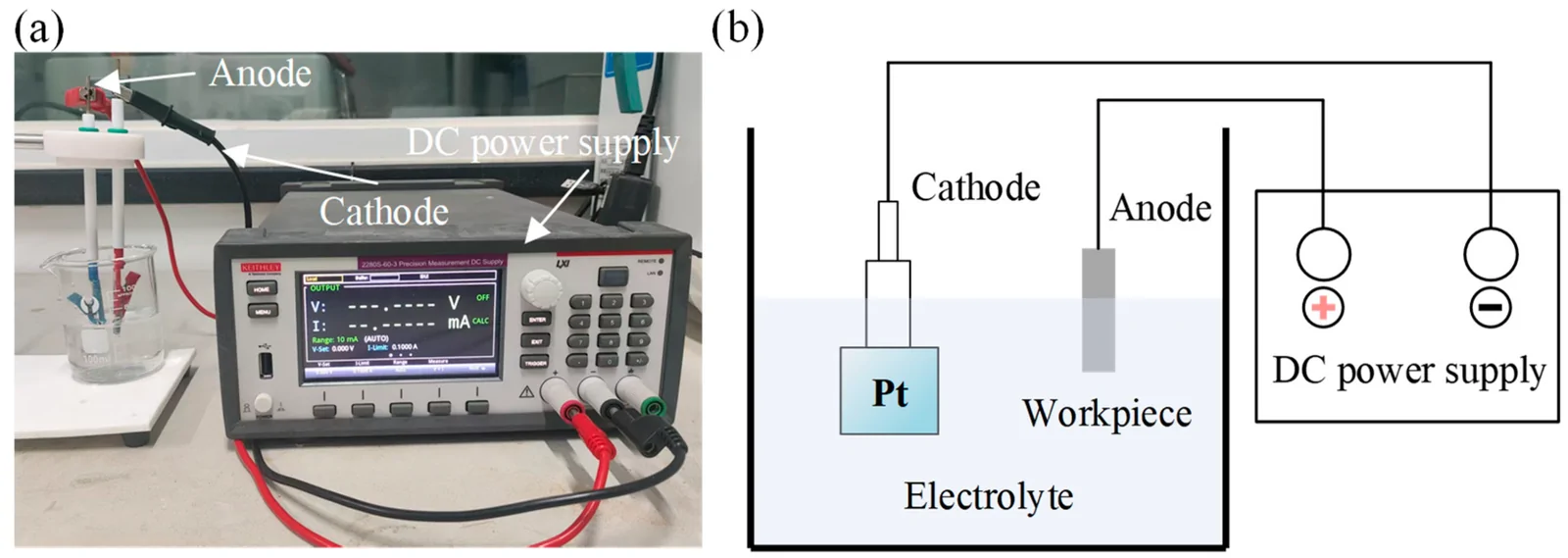
Experimental setup of electrochemical anodization: (**a**) photograph; (**b**) schematic diagram.

The anodic oxidation of Ti6Al4V in electrolytes containing fluoride ions can result in the formation of many types of dense oxide structures, including nanopores, nanotubes, and nanograss. In this study, a comprehensive analysis of single-factor experiments was carried out under consistent voltage circumstances. The objective was to examine the influence of oxidation voltage, ammonium fluoride (NH_4_F; analytical grade, Sinopharm Chemical Reagent Co., Ltd., Shanghai, China) concentration, and oxidation time on the quality of TiO_2_ nanotube fabrication. All anodization experiments were conducted in ethylene glycol (analytical grade, Sinopharm Chemical Reagent Co., Ltd., Shanghai, China) electrolytes containing the specified NH_4_F concentration and 2 vol% deionized water. Anodization was performed using a DC power supply (IT6723, ITECH Electronic Co., Ltd., Nanjing, China). Firstly, the process of anodic oxidation was conducted for a duration of 15 min, utilizing voltage settings of 40 V, 50 V, and 60 V. Subsequently, anodization was performed at a consistent voltage of 40 V for a duration of 15 min in ethylene glycol electrolytes with different NH_4_F concentrations of 0.5 wt.%, 0.7 wt.%, and 0.9 wt.%. Following that, anodization was carried out at 40 V for varying time intervals of 5 min, 15 min, and 30 min in an ethylene glycol electrolyte containing 0.5 wt.% NH_4_F and 2 vol% deionized water. Ultimately, a preliminary process of anodic oxidation was carried out for a duration of 10 min in an electrolyte solution consisting of ethylene glycol, with the addition of 0.5 wt.% NH_4_F and 2 vol% deionized water with voltage parameters of 40 V, 50 V, and 60 V. Following the first anodization, the samples were ultrasonically treated in deionized water for 45 min to remove the nanotube layer formed during the first anodization. The stripped Ti6Al4V substrate was then directly used for secondary anodization. The experimental setup is shown in Figure 1.

In summary, the experiments consisted of two parts: primary anodization was first used to investigate the effects of voltage, NH_4_F concentration, and anodization time, while secondary anodization was subsequently performed after ultrasonic removal of the nanotube layer formed during the first anodization. The same Ti6Al4V substrate after ultrasonic stripping was directly used for the secondary anodization.

### 2.2. TiO_2_ Nanotubes Fabrication Theory and Simulation

The development of TiO_2_ NTs through anodization is based on field-induced dissolution. Based on the growth-dissolution hypothesis, the electric field can stimulate both the oxidation and dissolution reactions, resulting in the formation of nanoscale hollow tube structures. This process comprises the essential reactions, as outlined in Equations (1) and (2).

(1) $$\mathrm{Ti}+2H_{2}O\to \mathrm{Ti}O_{2}+4H^{+}+4e^{-}$$

(2) $$\mathrm{Ti}O_{2}+6F^{-}+4H^{+}\to [\mathrm{Ti}F_{6}{]}^{2-}+2H_{2}O$$

**Figure 2:**
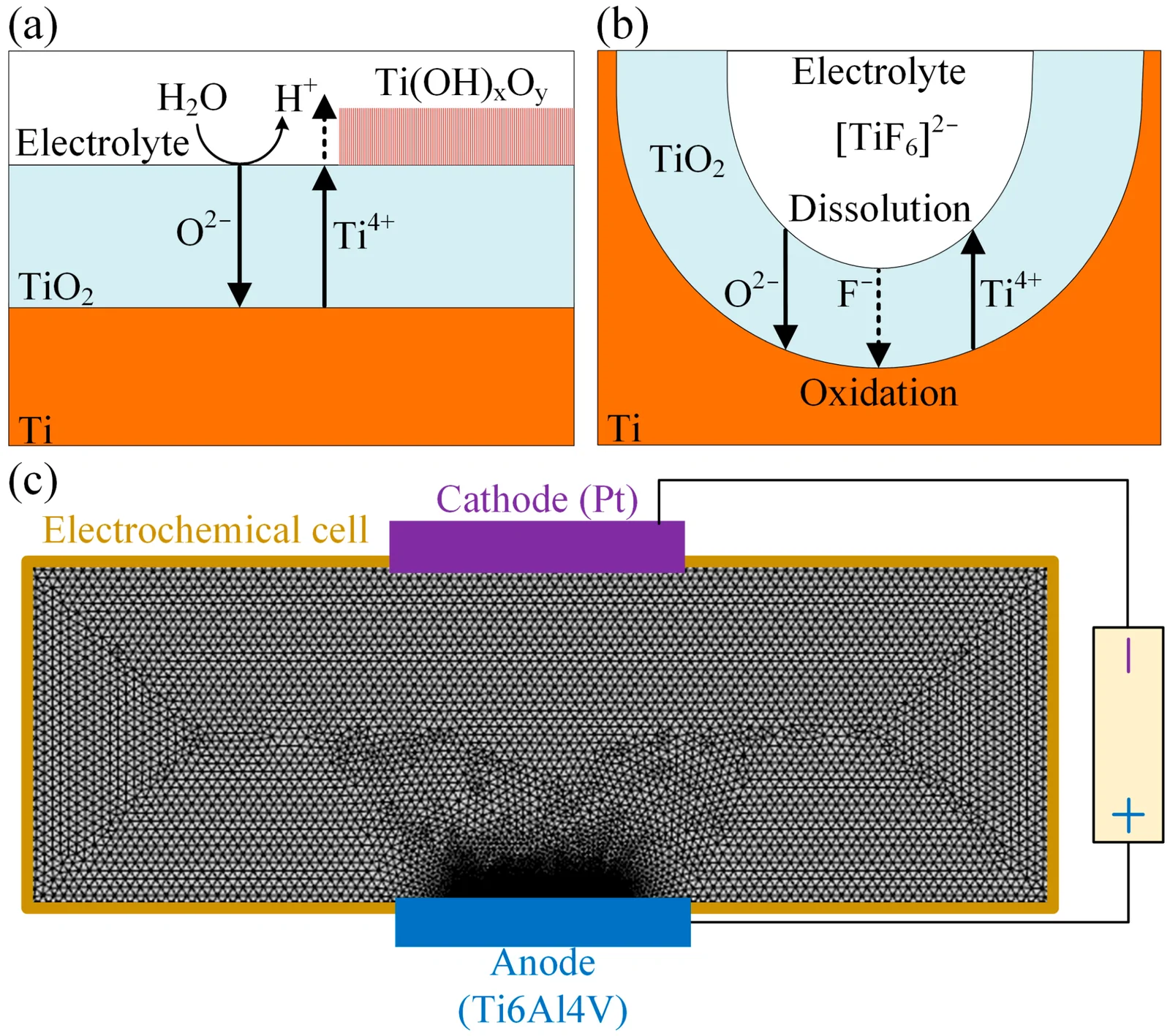
Dissolution mechanism and numerical modeling of TiO_2_ nanotube formation: (**a**) fluorine-free electrolyte; (**b**) fluorine-containing electrolyte; (**c**) COMSOL-based anodizing model.

The absence of fluorine ions (F^−^) in the electrolytes allows for the electric field to facilitate the movement of Ti_4_^+^ ions from the metal surface to the electrolyte surface. Simultaneously, negatively charged O_2_ ions migrate in the opposite direction, undergoing a reaction with the metal surface to produce a compact and stable layer of TiO_2_. The presence of a low-density hydroxide layer of Ti(OH)_x_O_y_ complements this layer. Figure 2a illustrates the cross-sectional structure with many layers. On the other hand, in fluorine electrolytes, the F^−^ ions have a crucial function by starting the process of dissolving and forming complex compounds within the compact oxide layer. The contact triggers the activation of non-uniform micropores on the oxide layer surface, as depicted in Figure 2b. As the thickness of the oxide increases, the small openings expand and become interconnected, resulting in the spontaneous development of structures resembling nanotubes. The development of these structures is impacted by the stable environmental conditions and the continuous flow of electric current in the electrolyte, which encourages their arrangement in the direction of the electric field.

The isoelectric field theory, although sharing similarities with the field-induced dissolution theory in its dependence on the growth-dissolution hypothesis, proposes a significant distinction: nanotubes can grow within a distinct fan-shaped region along the direction of the electric field, rather than exclusively along the normal direction of the tube length. The rate of oxide layer formation is controlled by the current density, which is focused on the edges of the oxidized area, resulting in uneven thickness of the oxide.

The latest progress in the field questions the indispensability of F^−^ ions in the fabrication of anodized TiO_2_ NTs. According to the viscous flow theory, the flow of oxides is facilitated by internal stresses caused by volume expansion and ion conductivity. This theory has resulted in a precise computational model for determining the thickness of layers in anodized TiO_2_ NTs. It is suggested that the avalanche electron current resulting from dielectric breakdown decreases the number of O_2_ ions in the oxide layer, causing the formation of oxygen bubbles that serve as molds. The bubbles undergo expansion due to the pressure exerted by the electrolyte and then interact with the adjacent oxides. Ultimately, the particles migrate towards the electrolyte, undergoing expansion and rupture to create even thick hemispherical cavities, which then develop into porous nanotubes.

To provide mechanistic support for the experimental observations, COMSOL Multiphysics has been utilized to build up the anodizing model for titanium alloy. The methodology employed entails building a model that relies on particular assumptions to examine the present and potential spread, as well as the rate at which the dense oxide layer forms during anodization. This analysis assumes that the electric field is evenly distributed and does not consider any impacts related to the boundaries in areas that have not been processed. The electrolyte composition remains constant, hence simplifying intricate chemical reactions into electron transfer processes. An additional assumption is also made that the electrolyte concentration is both isotropic and uniform, disregarding any convective effects that may occur throughout the reaction.

The simulation model, as depicted in Figure 2c, utilizes a triangular mesh that is not constrained for the electrolyte area, which is enclosed by a 10 mm × 30 mm insulating electrochemical cell. The electrolyte, a low-conductivity organic solvent, is in contact with anode and cathode electrodes constructed of Ti6Al4V and Platinum (Pt), respectively, measuring 1 mm × 10 mm. The Ti6Al4V workpiece itself was defined as the anode, while the Pt electrode served as the cathode. Therefore, the model does not represent an additional sample placed between the anode and cathode. The electrolyte domain represents the region between the two electrodes. The electrode potentials are directly implemented, with localized mesh refinement at the anode reaction site, utilizing parameters from COMSOL library.

The simulation procedure is divided into two stages: the first stage involves initializing the current distribution, while the second stage focuses on geometric deformation. At first, the steady-state equation solves the initial distribution of the potential field. Subsequently, this result is utilized in the transient equation to simulate the process of electrode alteration.

The control equations governing the behavior of the electrolyte in the secondary current distribution model are based on the principles of current density conservation and Ohm’s law. The equations involved include the electrolyte’s total mass balance equation (Equation (3)), the conservation of current density (Equation (4)), and the rate at which the anode dissolves (Equation (5)). Anode kinetics are integrated by utilizing experimental polarization curves available in COMSOL Multiphysics’s library.

(3) $$\nabla \cdot N_{i}=\nabla \cdot \left(-D_{i}\nabla c_{i}-z_{i}m_{i}Fc_{i}\nabla \Phi_{l}+c_{i}u\right)=0$$

(4) $$\nabla \cdot I=\nabla \cdot \left(-\sigma_{l}\nabla \Phi_{l}\right)=0$$

(5) $$V_{d}=-\frac{R_{d}\eta F}{I_{loc}}$$

The equations incorporate various parameters such as substance flux (*N_i_*), diffusion rates (*D_i_*), ion concentration (*c_i_*), valence (*z_i_*), ion mobility (*m_i_*), Faraday constant (*F*), ionic potential (*Φ*_*l*_), velocity vector (*u*), current density (*I*), electrolyte conductivity (*σ_l_*) ohmic resistance (*R_d_*), equilibrium potential (*η*), and local anode current density (*I_loc_*). These components together enable a thorough comprehension of the electrochemical dynamics involved in the anodic oxidation process.

### 2.3. Characterization and Measurement

The morphological and topographical properties of the surfaces were investigated with the assistance of a field-emission scanning electron microscope (SEM, S-4800, Hitachi, Tokyo, Japan), while an acceleration voltage of 15 kV was utilized. Because no phase- or composition-specific characterization was performed in the present study, the SEM observations were used primarily to evaluate the morphology of the anodic nanotubular oxide structures. The assignment of these structures to TiO_2_-based anodic oxides follows the established anodization behavior of Ti-based substrates in fluoride-containing electrolytes reported in previous studies. Each of the substrates was subjected to ultrasonic cleaning for a period of 10 min at a temperature of 20 °C using ethanol (C_2_H_5_OH). This was done in order to guarantee that the substrates were clean. The current-density curves presented in this study correspond to individual anodization measurements rather than averaged curves from independent replicate experiments. The current density was calculated by normalizing the measured current to the exposed anodic surface area, which was kept constant for all experiments. COMSOL Multiphysics’s electrochemistry module was employed to model the anodic oxidation process of Ti6Al4V materials in this study. This module is renowned for its accuracy and flexibility, making it extensively utilized in simulations for many applications, such as fuel cell design and metal corrosion prevention. The module includes interfaces for analyzing primary, secondary, and tertiary current distributions. It considers factors such as electrolyte conductivity, mass transfer of ions and reactants, and electrode kinetics. The analysis focuses on the potential distribution, current conduction, and particle transport in the electrolyte.

## 3. Results and Discussion

### 3.1. Electrochemical Simulation Results

**Figure 3:**
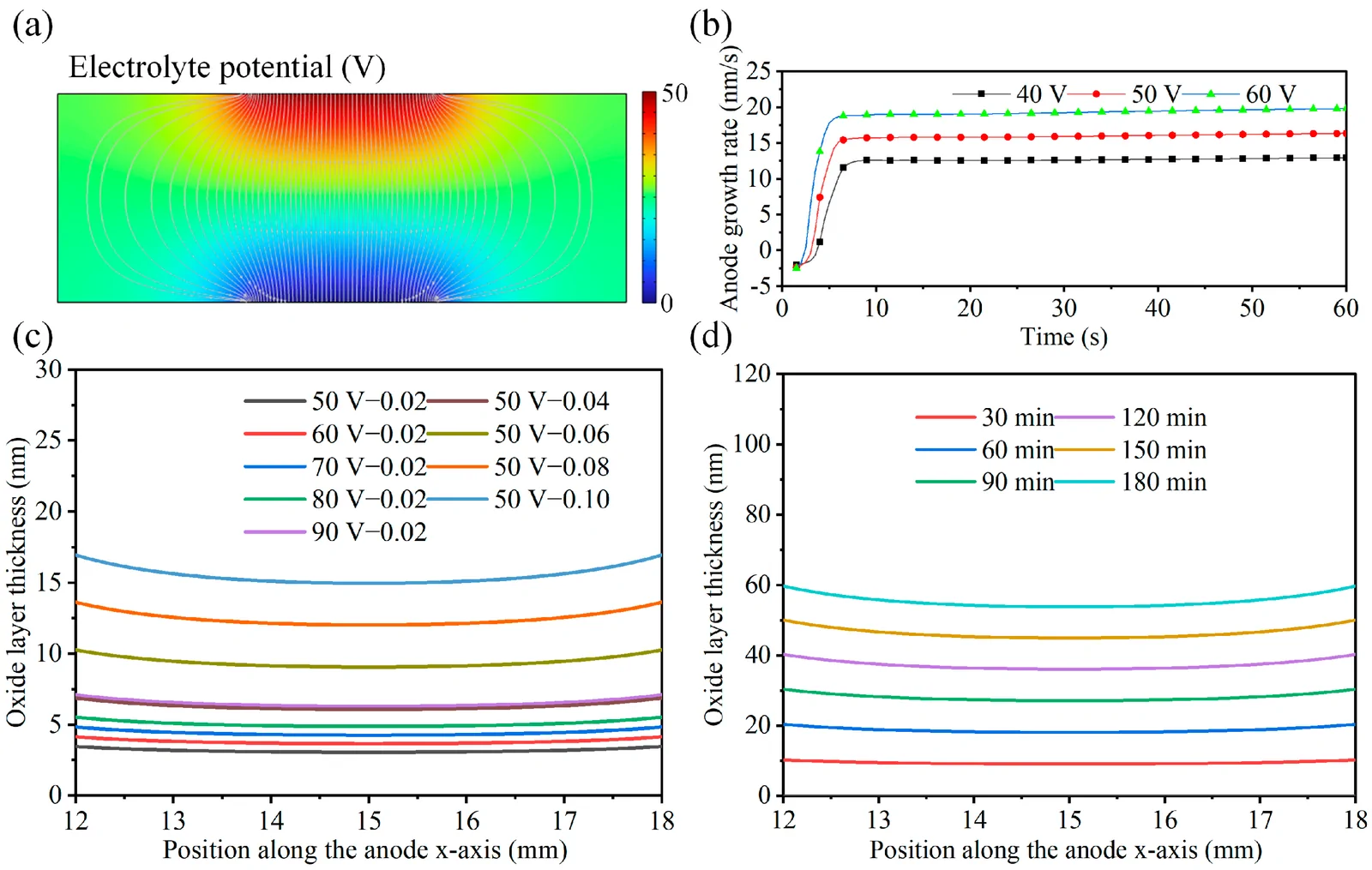
Simulation results and oxide layer evolution under different conditions: (**a**) potential and current density at 50 V and 0.21 S/m; (**b**) anode growth rate; (**c**) oxide morphology after 10 min under varied parameters; (**d**) linear growth of the oxide layer.

The simulation results provide mechanistic support for understanding the effects of anodization voltage, electrolyte conductivity, and oxidation time on the anodization process for the formation of TiO_2_ NTs. Figure 3a demonstrates the 2D electric field distribution model of under the voltage of 50 V. The electric potential is indicated by the covers ranging from 50 V (Red) to 0 V (Blue). The current density vectors exhibit a fan-like distribution between the electrodes, indicating that the macroscopic current field remains relatively stable under the adopted boundary conditions. As shown in Figure 3b, the anode growth rate increases rapidly at the initial stage and then gradually stabilizes. A higher anodization voltage promotes oxide growth by enhancing field-driven ion migration, but excessive voltage may intensify local current density concentration and increase structural instability.

Figure 3c,d shows the plotted thickness of the uniform oxide layer resulting from simulations of several parameters, including varying oxidation voltages, electrolyte conductivities, and oxidation times. The x = 12–18 mm interval was selected because it corresponds to the central region of the anodic surface, where the influence of electrode-edge effects is relatively small. The same spatial interval was used for all conditions to ensure a consistent comparison. Figure 3c illustrates the combined influence of voltage and electrolyte conductivity on the thickness of the oxide layer. As the voltage or electrolyte conductivity rises, the thickness of the oxidation layer also increases, and the uneven distribution at the edges becomes more noticeable. For example, under the condition of 50 V–0.02 S/m, the simulated oxide-layer thickness is approximately 3.24 μm. Because systematic cross-sectional thickness measurements were not performed in the present study, this simulated value is used only to illustrate the predicted oxide-growth trend rather than as a quantitatively validated experimental thickness. Therefore, the simulation results are primarily used to provide qualitative insight into oxide-growth trends under different anodization conditions.

Furthermore, as depicted in Figure 3d, the thickness of the oxide layer is comparable under conditions of 90 V–0.02 S/m and 50 V–0.04 S/m. However, in practical studies, it has been observed that high voltage can result in fluctuations in potential and the breakdown of the anode. Modifying the ratio of conductive components in the organic solvent can effectively reduce these problems by adjusting the electrolyte conductivity. Hence, it is advisable to initially optimize the electrolyte conductivity prior to contemplating voltage adjustments as a means of regulating the thickness of the anodic oxidation layer.

Figure 3d illustrates the development of the oxide layer under conditions of 50 V–0.02 S/m. It demonstrates that the thickness of the oxide layer increases at a nearly constant rate over time. As per the field-induced dissolution theory, the electric field causes the anode surface to quickly develop a compact oxide layer. Upon reaching a specific thickness, the oxide layer undergoes dissolution, resulting in the formation of micropores. Over time, this process achieves a state of equilibrium known as generation-dissolution, leading to the fabrication of TiO_2_ NTs. The simulation, although excluding alterations in chemical reactions, offers a decent estimate for the stable equilibrium phase, providing substantial insight into the anodization process.

**Figure 4:**
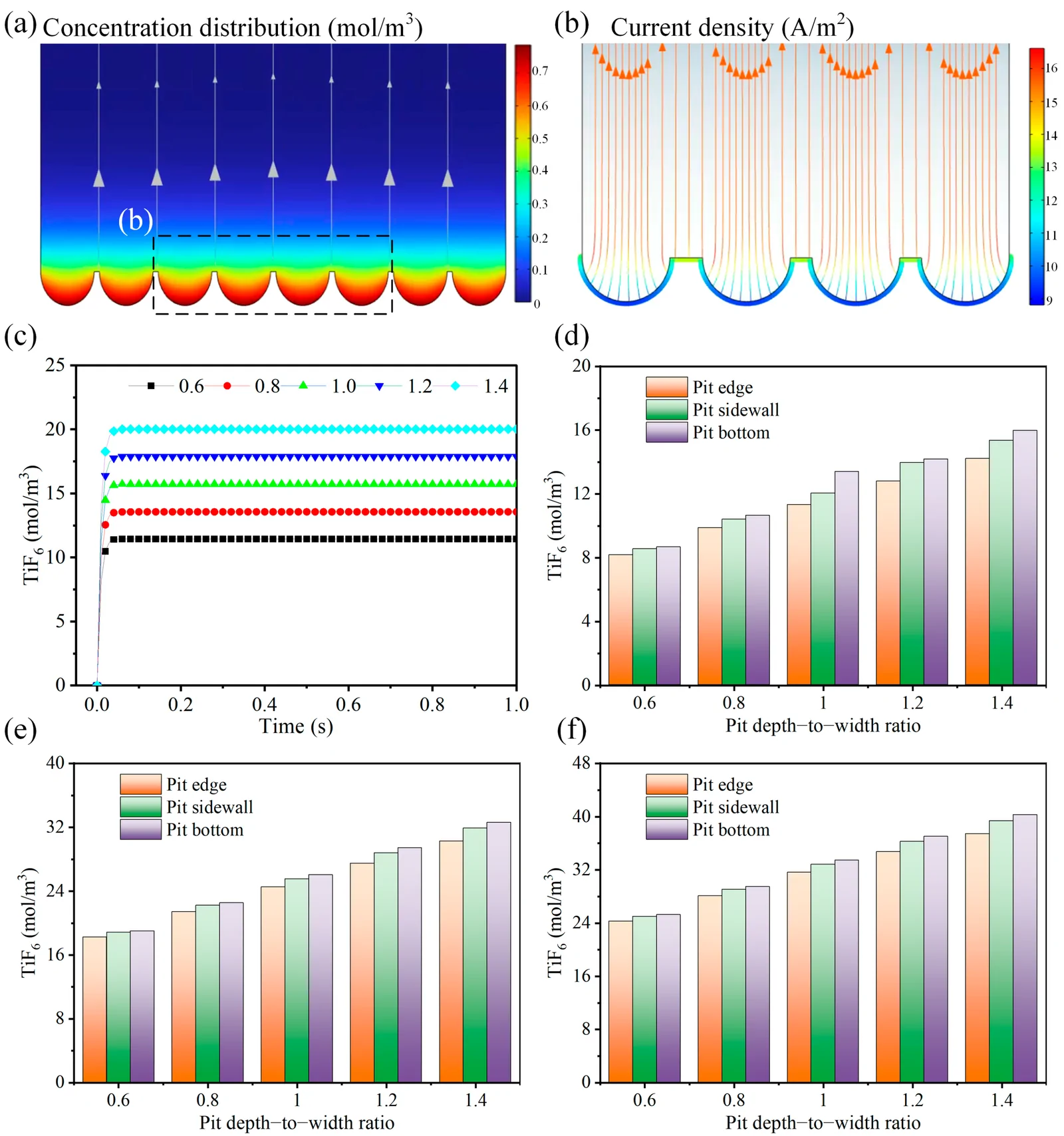
Mass transfer and current-density distribution during secondary anodization on pre-pitted Ti6Al4V substrates: (**a**) TiF_6_^2−^ concentration distribution; (**b**) current-density distribution on the anodic surface; (**c**) TiF_6_^2−^ concentration at the pit bottom under different pit depth-to-width ratios; (**d**–**f**) TiF_6_^2−^ concentration at the pit rim, sidewall, and bottom under different pit depth-to-width ratios at 40, 50, and 60 V, respectively.

To further investigate the effect of preformed pits on secondary anodization, a mass-transfer-coupled electrochemical model was established based on a pre-pitted Ti6Al4V substrate. The pit diameter was fixed at 100 μm, and different pit depth-to-width ratios were considered. This pit size was adopted as an idealized computational geometry for examining the qualitative effects of pit geometry on local current-density and mass-transfer behavior and does not represent the experimentally measured dimensions of individual nanotubes or surface pits. TiF_6_^2−^ was selected as the representative soluble titanium-fluoride complex generated during fluoride-assisted dissolution. As shown in Figure 4a, TiF_6_^2−^ tends to accumulate inside the preformed pits, especially near the lower region of the pit. This indicates that the pit geometry restricts the outward diffusion of soluble products. Figure 4b shows that the current density on the anodic surface is nonuniformly distributed along the curved pit surface, suggesting that the preformed pits can modify the local electrochemical reaction during secondary anodization.

Figure 4c shows the time-dependent TiF_6_^2−^ concentration at the pit bottom under different pit depth-to-width ratios. The concentration increases rapidly at the initial stage and then reaches a quasi-steady state, indicating that the generation and diffusion of soluble products quickly approach a dynamic balance. With increasing pit depth-to-width ratio, the steady-state TiF_6_^2−^ concentration at the pit bottom increases gradually. This can be attributed to the longer diffusion path and stronger geometric confinement in deeper pits, which make it more difficult for soluble products to diffuse from the pit bottom to the bulk electrolyte.

The local TiF_6_^2−^ concentration at different pit positions under 40, 50, and 60 V is shown in Figure 4d–f. The TiF_6_^2−^ concentration exhibited a spatially nonuniform distribution within the pit, showing pronounced enrichment near the pit bottom and gradually decreasing toward the pit sidewall and edge. Meanwhile, the TiF_6_^2−^ concentration increases with both the pit depth-to-width ratio and the applied voltage. These results suggest that deeper pits and higher voltages promote the accumulation of soluble titanium-fluoride complexes, which may enhance local chemical dissolution at the pit bottom. Therefore, the simulation indicates that secondary anodization is affected by the coupling of local current-density redistribution and diffusion-limited dissolution inside the preformed pits.

The present COMSOL model was developed to clarify the coupled electrochemical and mass-transfer behavior during anodization, particularly the influence of preformed pits on secondary anodization. In the voltage-conductivity-time simulations, the model describes the potential distribution, current-density distribution, and oxide-layer evolution under different anodization conditions, providing insight into the effect of electrical parameters on oxide growth. In the pre-pitted substrate model, TiF_6_^2−^ was introduced as a representative soluble titanium-fluoride complex generated during fluoride-assisted dissolution. The simulation shows that TiF_6_^2−^ tends to accumulate inside the pits, especially near the pit bottom, while the local current density is redistributed along the curved pit surface. These simulation results indicate that, if preformed pits are present after ultrasonic removal of the first anodic layer, they may influence secondary anodization through geometric confinement, diffusion-limited mass transfer, and localized electrochemical reactions. Although the model does not explicitly resolve all microscopic processes involved in TiO_2_ nanotube self-organization, such as local pH variation, oxide stress evolution, viscous oxide flow, oxygen-bubble dynamics, and nanotube wall separation, it provides useful mechanistic support for understanding current-density redistribution, TiF_6_^2−^ accumulation, and localized oxide growth during secondary anodization.

Therefore, the present model should be regarded as a phenomenological model for interpreting current-density redistribution and mass-transfer behavior rather than a predictive model of nanotube nucleation, wall separation, oxide-stress evolution, or nanograss formation.

### 3.2. Current Density Variation

The anodization process parameters have a significant impact on the variation curves of current density. Throughout the oxidation process, the mean output voltages were measured to be 39.9982 V, 49.9991 V, and 59.9997 V, exhibiting a small fluctuation rate of 0.0023%. This suggests a consistent and dependable output.

**Figure 5:**
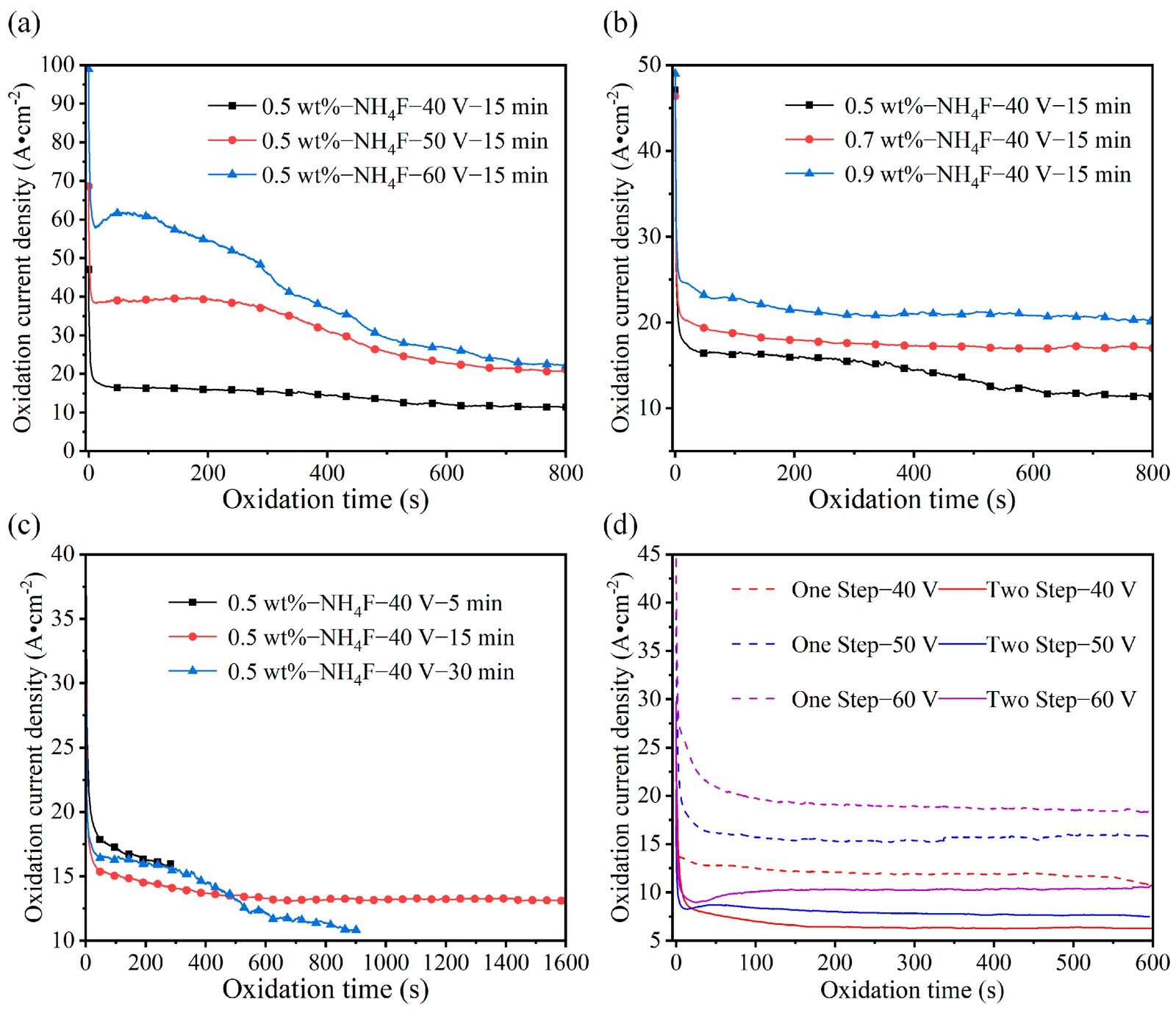
Current density-time curve during anodization process: (**a**) oxidation process under different voltages, (**b**) oxidation process under different fluoride concentrations, (**c**) oxidation process under different oxidation times, (**d**) current comparison diagram of primary/secondary oxidation.

Figure 5a exhibits the current density-time curves at different voltages, showing the characteristic evolution of the anodization current, including an initial current decay, a subsequent transition or recovery stage, and a later quasi-steady stage.

To provide a quantitative comparison of the current-density curves, the peak current density (*J*_peak_), minimum current density after the initial decay (*J*_min_), and steady-state current density (*J*_ss_) were extracted, as summarized in Table 1. *J*_peak_ was defined as the maximum recorded current density, *J*_min_ as the minimum current density identified after the initial current decay, and *J*_ss_ was calculated as the average current density over the final 10% of the recorded anodization period.

In the initial phase, there is a notable decline in the amount of electric current, which is caused by the quick development of a compact layer of oxide on the substance. This leads to an elevated level of resistance and a substantial drop in the flow of electric current. In the subsequent stage, the current may partially recover or undergo a transition depending on the applied voltage, which can be associated with changes in the compact oxide layer and the development of conductive pathways. In the last step, there is a progressive decrease in the current strength until it reaches a stable value. This indicates the continued growth of TiO_2_ NTs, a uniform distribution of the oxidation layer, and eventual stabilization in a balanced condition. Increasing the oxidation voltages leads to a higher current density and a more prominent current revival in the second stage. This behavior may be associated with enhanced local breakdown of the compact oxide layer at higher applied voltages, facilitating the subsequent electrochemical evolution of the anodic surface. At lower voltages, the current response may also be influenced by the heterogeneous electrochemical behavior of the alloying constituents in Ti6Al4V. However, the specific chemical states of Al- and V-containing species in the anodic layer were not characterized in the present study, and therefore no definitive compositional interpretation is made here.

As the anodization voltage increased from 40 to 60 V, *J*_peak_ increased from 47.09 to 98.98 A·cm^−2^, while *J*_ss_ increased from 11.26 to 21.89 A·cm^−2^. Meanwhile, the nanotube outer diameter increased from 75.76 to 124.84 nm. These results quantitatively support the observation that a stronger electrochemical response at higher applied voltage is accompanied by an increase in nanotube diameter and more pronounced morphological evolution.

Figure 5b depicts that higher levels of fluoride in the electrolyte result in a greater current density. The growth-dissolution rate of the oxides is influenced by the varied concentrations of F^−^ ions, which are linked to varying chemical equilibrium states. Variation in oxidation time Figure 5c indicates negligible variations in the magnitude of current changes as oxidation time varies, demonstrating a consistent overall pattern. The modest variances occur due to variations in surface conditions and roughness among the samples. Figure 5d demonstrates that, when subjected to the same process conditions, the current density during the second anodization is lower compared to the first oxidation. Additionally, there is a more noticeable revival in the second stage. The more pronounced current recovery during secondary anodization may be associated with the altered initial surface state after removal of the first anodic layer. Oxygen-bubble-assisted growth has been proposed in the literature as one possible mechanism for anodic nanotube formation; however, no direct evidence of bubble-controlled growth was obtained in the present study. Therefore, the oxygen-bubble mechanism is considered only as a possible interpretation rather than a confirmed growth mechanism. However, oxygen-bubble dynamics were not directly characterized in the present study; therefore, this mechanism is considered a possible interpretation rather than direct experimental evidence.

### 3.3. Morphology Characterization

**Figure 6:**
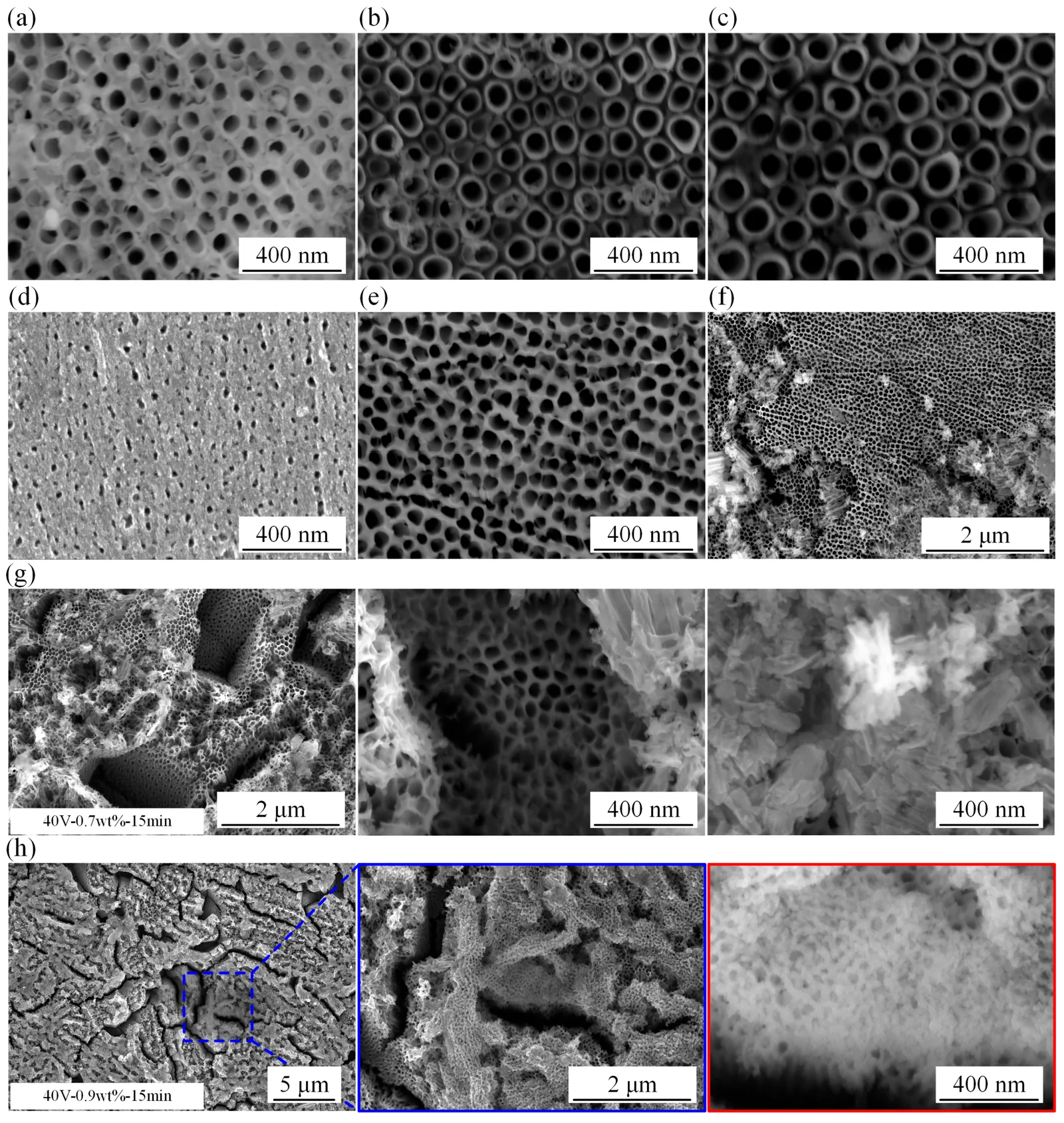
Influence of oxidation parameters on surface and nanotube morphology: (**a**–**c**) surface morphology at 0.5 wt.% electrolyte for 15 min under 40 V, 50 V, and 60 V; (**d**–**f**) nanotube morphology at 0.5 wt.% and 40 V for 5 min, 15 min, and 30 min; (**g**,**h**) surface morphology at 40 V and 15 min under electrolyte concentrations of 0.7 wt.%, and 0.9 wt.%.

The impact of process factors on the quality of TiO_2_ nanotubes is clearly illustrated in Figure 6. The figure shows the different shapes and structures of nanotubes that were produced during a primary oxidation experiment conducted under different conditions. Figure 6a–c depict nanotubes that were developed using oxidation voltages of 40 V, 50 V, and 60 V, respectively. As the voltage increases, the nanotube structures become more distinct and fully formed. The increase in voltage causes the walls of the nanotubes to erode at a faster pace, resulting in their overall growth. Specifically, the outer diameter of the nanotubes increases from 75.76 nm to 93.3 nm and 124.84 nm, respectively. This voltage-dependent increase in nanotube diameter is consistent with previous studies, which generally reported that increasing anodization voltage promotes the formation of larger nanotube diameters. The nanotube morphology seen at various oxidation periods is consistent with the theoretical predictions of their growth. Extended anodization durations resulted in progressive changes in the surface morphology of the nanotubular structures. After 5 min, the material surface exhibits the initial development of nanotubes, as evidenced by the presence of microcracks and micropits, as shown in Figure 6d. Nanotube structures resembling those depicted in Figure 6a are generated after a duration of 15 min. Prolonging the oxidation duration to 30 min results in the amalgamation of neighbouring nanotubes into structures resembling lotus roots, which are denser and more stable, as illustrated in Figure 6e. Prolonged anodization may enhance the competition between oxide formation and fluoride-assisted dissolution, resulting in progressive deterioration of the surface morphology. This imbalance may contribute to the formation of nanograss-like structures, as shown in Figure 6f.

As the concentration of fluoride in the electrolyte increases, the collapse of the surface layer becomes more noticeable. When the NH_4_F concentration is 0.7 wt.%, the substrate surface continues to produce nanotubes that are mostly transparent. However, there is a significant presence of nano-grass fibers at the tips, as depicted in Figure 6g. When the concentration of NH_4_F is 0.9 wt.%, the adhesion between nanotubes becomes stronger, resulting in a noticeable change in their shape. Figure 6h shows that the surface is characterized by a compact oxide layer with uneven cracks at the micrometer level, which then transforms into smooth, porous layers at the nanoscale level. This tendency is consistent with previous reports showing that excessive fluoride concentration enhances chemical dissolution of the anodic oxide and can promote surface damage and nanograss formation.

**Figure 7:**
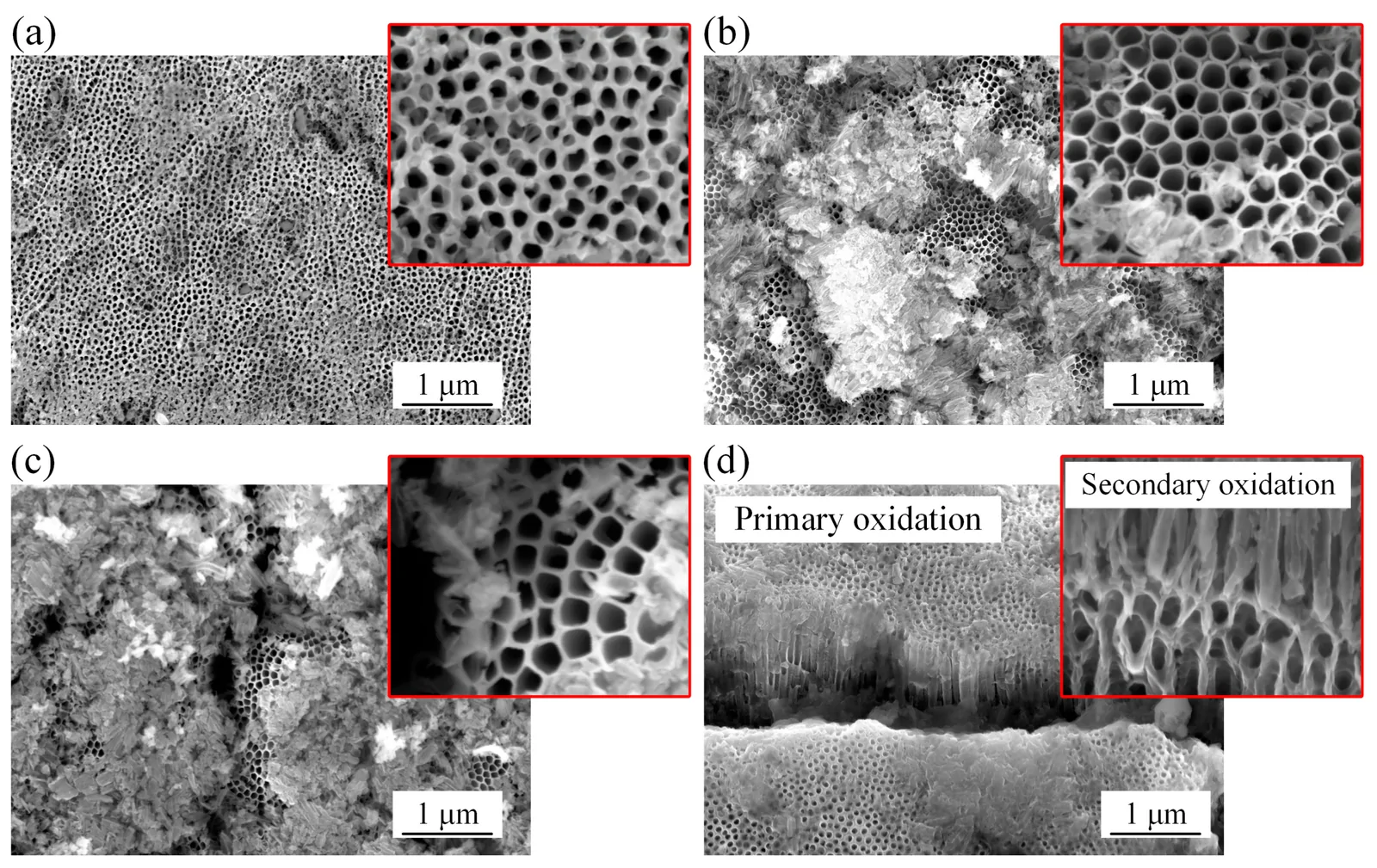
Nanotube morphology under secondary oxidation: (**a**) secondary oxidation voltage 40 V, (**b**) secondary oxidation voltage 50 V, (**c**) secondary oxidation voltage 60 V, (**d**) lateral morphology of primary/secondary oxidation.

Oxidation process that occurs after the initial oxidation. Nanotube Structure. Figure 7 depicts the structure of nanotubes that are created through secondary oxidation using various process parameters. The representative outer diameters measured from the SEM images were approximately 48.87 nm, 58.46 nm, and 72.99 nm at 40 V, 50 V, and 60 V, respectively, which were smaller than the corresponding values observed after primary anodization. These measurements indicate a tendency toward smaller nanotube diameters after secondary anodization. However, because independently fabricated replicate specimens and a predefined statistical sampling protocol were not included in the original experimental design, these diameter values should be regarded as representative morphological measurements rather than statistically validated population averages. Therefore, the comparison is used primarily to describe the observed morphology evolution between primary and secondary anodization. The change in the surface state after ultrasonic removal of the first nanotube layer may influence the local current-density distribution and subsequent nanotube growth during secondary anodization.

When the voltage is set at 40 V, the secondary oxidation process leads to a more even and polished surface of oxidation, as depicted in Figure 7a. Nevertheless, as the voltage reaches 50 V and 60 V, the surface becomes completely coated with a thick layer of densely packed nanograss, as depicted in Figure 7b,c. Greater oxidation voltage results in a more concentrated dispersion of nanograss. The main cause of this phenomenon is the heightened chemical reactions that occur at higher voltages. The observed morphology may be associated with changes in the initial surface state after removal of the first nanotube layer. If pit-like surface features are formed during this process, they may influence local current-density distribution and subsequent anodization behavior. Moreover, increased voltages in secondary oxidation have a greater probability of penetrating the compact oxide layer and creating conductive pathways, facilitating the electrolyte’s access to the base of the nanotubes. This outcome is consistent with the patterns found in the current strength-time curves. At 50 V, the nanotubular regions exhibited relatively regular circular openings compared with the lotus-root-like and square-opening morphologies observed at 40 V and 60 V, respectively, as depicted in Figure 7d. However, nanograss was also observed on the surface at 50 V.

**Figure 8:**
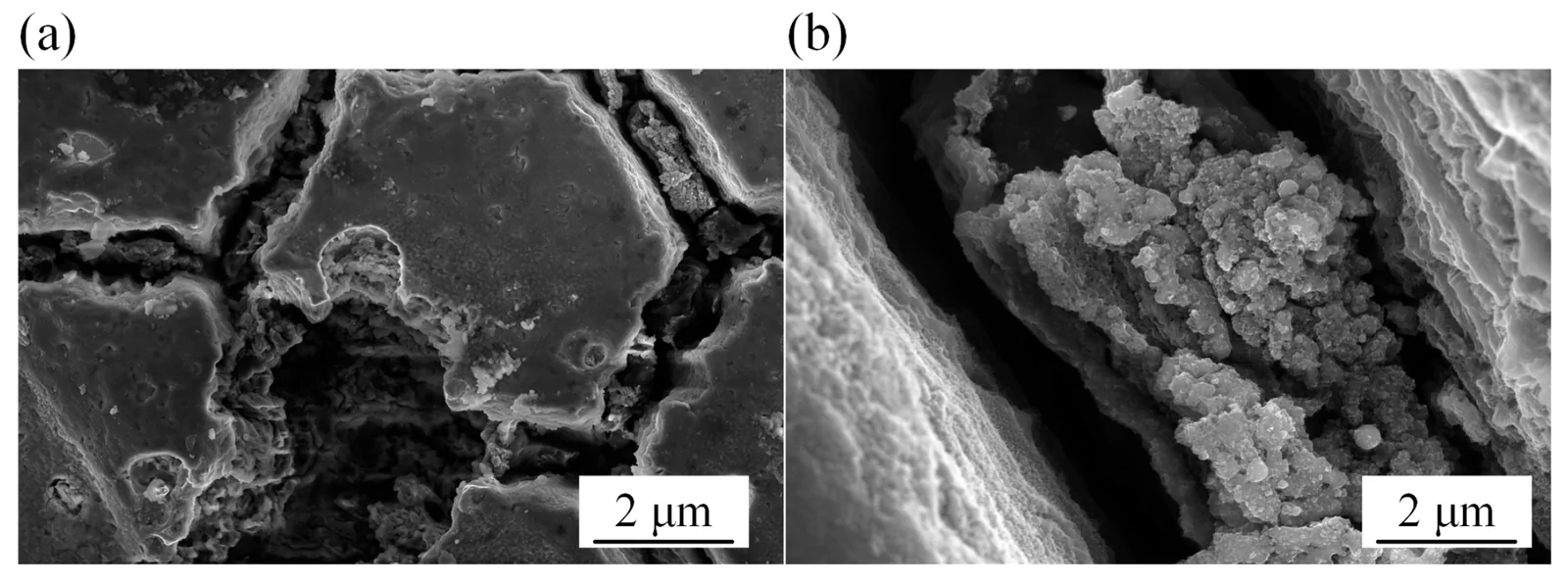
Damage to the surface substrate of multi-level structures caused by anodizing process: (**a**) hexagonal prism broken down under excessive voltage conditions, (**b**) trench deposits under excessive oxidation time.

For the comparison shown in Figure 7d, half of the Ti6Al4V surface was masked with insulating tape during the first anodization. After the first anodization, the nanotube layer was removed by ultrasonic treatment. The insulating tape was then removed, and the sample was subjected to secondary anodization. This procedure produced adjacent regions with different anodization histories on the same specimen. The nanotubes that are produced during the initial anodic oxidation display a more uniform, dense, and perpendicular arrangement. In contrast, the nanotubes made during the subsequent anodic oxidation entangle with each other, resulting in intricate structures that are clearly distinct from the morphology observed in the first oxidation. Among the conditions investigated in this study, primary anodization at 40 V followed by secondary anodization at 50 V produced relatively regular nanotubular regions, although nanograss was also present on the surface. Therefore, this condition is considered comparatively favorable only in terms of the observed nanotubular morphology and should not be regarded as a globally optimized process window. In addition, representative microscale substrate damage was observed under excessive anodization voltage and prolonged anodization time, as shown in Figure 8, indicating that severe anodization conditions may adversely affect the underlying microstructures.

The morphology difference between primary and secondary anodization may be related to changes in the initial surface state. During primary anodization, nanotube formation starts from the original Ti6Al4V surface, whereas the second anodization is performed after ultrasonic removal of the first anodic layer. This removal process may alter the surface topography and produce pit-like surface features, which could affect the local electric-field and current-density distributions during subsequent anodization. However, the dimensions, density, and roughness of these surface features were not directly quantified in the present study; therefore, their role is considered a possible mechanistic interpretation rather than direct experimental evidence.

## 4. Conclusions

This study investigated the influence of anodization voltage, fluoride ion concentration, oxidation time, and secondary anodization on the morphology evolution of anodic nanotubular oxide structures formed on Ti6Al4V substrates. The conclusions deducted from this study can be summarized as follow:

(1) Within the investigated voltage range, anodization voltage showed a clear influence on TiO_2_ nanotube morphology. Increased voltages result in more distinct and comprehensive nanotube formations. The faster erosion rate of nanotube walls under larger voltages leads to the expansion of the nanotubes and an increase in their outer diameter.
(2) The time duration of the oxidation process and the concentration of fluoride ions in the electrolyte are important factors that significantly influence the quality and structure of the nanotubes. Extended oxidation durations lead to progressive changes in nanotubular surface morphology, with potential structural deterioration such as nanograss formation and tube collapse. Elevated levels of fluoride even can lead to a more pronounced deterioration of the surface oxidation layer and have a substantial impact on the shape of the nanotubes, sometimes causing them to collapse into flat, porous layers.
(3) Varied process conditions result in secondary oxidation, which causes a decrease in the outer diameter of the nanotubes in comparison to initial oxidation. Increased oxidation voltages during secondary oxidation led to a more concentrated arrangement of nanograss on the surface. This phenomenon may be related to changes in the initial surface state after ultrasonic removal of the first anodic layer and the associated local current-density redistribution.
(4) Among the investigated conditions, primary anodization at 40 V in 0.5 wt.% NH_4_F electrolyte followed by secondary anodization at 50 V produced relatively regular nanotubular regions, although nanograss was also present on the surface. Further experiments over a broader parameter range would be required to establish an optimized process window.

Overall, secondary anodization after ultrasonic removal of the first nanotube layer provides an additional route for regulating TiO_2_ nanotube morphology on Ti6Al4V substrates. However, further functional evaluations are required to determine its application-specific advantages.

## Figures and Tables

**Table 1:** Quantitative parameters extracted from the current density-time curves at different anodization voltages.

Voltage (V)	*J*_peak_ (A·cm^−2^)	*J*_min_ (A·cm^−2^)	*J*_ss_ (A·cm^−2^)	Nanotube Outer Diameter (nm)
40	47.09	16.26	11.26	75.76
50	68.63	38.36	20.85	93.30
60	98.98	58.03	21.89	124.84

## Data Availability

The data presented in this study are available on request from the first author.
